# GlyT1 (SLC6A9) inhibition in neurological and psychiatric disorders

**DOI:** 10.1007/s00210-026-05276-y

**Published:** 2026-04-14

**Authors:** Daniel Pereira Cavalcante, Gustavo Almeida Carvalho, Antônio Ítalo Santos Nunes, Amanda Rodrigues Quintanilha, Lucas Rodrigues Couto Nascimento, Leonardo Caixeta, Henning Ulrich, Renato Santiago Gomez, Mauro Cunha Xavier Pinto

**Affiliations:** 1 Department of Pharmacology, Institute of Biological Sciences, Federal University of Goiás, Goiânia, GO Brazil; 2 School of Medicine, Federal University of Goiás, Goiânia, GO Brazil; 3 Institute of Chemistry, University of São Paulo, São Paulo, SP Brazil; 4 School of Medicine, Federal University of Minas Gerais, Belo Horizonte, MG Brazil

**Keywords:** Glycine transporter type 1, SLC6A9, Excitotoxicity, Schizophrenia, Parkinson’s disease, Alzheimer’s disease, Stroke

## Abstract

Glycine is a fundamental neuroactive amino acid that serves dual roles in the central nervous system: acting as a primary inhibitory neurotransmitter via strychnine-sensitive glycine receptors and as an essential co-agonist at the N-methyl-D-aspartate (NMDA) receptor. This dual functionality is important for maintaining the excitation–inhibition balance, synaptic plasticity, and network stability. The spatial and temporal availability of glycine is strictly regulated by two high-affinity, Na^+^/Cl^−^-dependent transporters: GlyT1 (SLC6A9) and GlyT2 (SLC6A5). These transporters exhibit distinct cellular distributions and functional specializations. GlyT1 is predominantly expressed in astrocytes and specific neuronal populations, where it buffers ambient glycine levels to modulate NMDA receptor activity. In contrast, GlyT2 is primarily localized to presynaptic terminals of glycinergic neurons, where it facilitates vesicular refilling essential for inhibitory signaling. This review provides a comprehensive overview of glycine metabolism, the structural biology and transport cycles of SLC6 glycine transporters, and the neuroanatomical framework of GlyT1 function. We further synthesize pharmacological advances in GlyT1 inhibition, evaluating both sarcosine-derived and non-sarcosine inhibitors, such as NFPS (ALX-5407), bitopertin, and iclepertin. The clinical and preclinical evidence for GlyT1 as a therapeutic target in psychiatric, neurological, and neurodegenerative disorders is critically assessed. Finally, we address key translational challenges, including dosing constraints, compensatory mechanisms, and SLC6 family selectivity, while highlighting the potential of structure-guided design to refine GlyT1-targeted therapies.

## Neurobiology of L-glycine

Glycine is one of the smallest proteinogenic amino acids and a key component of proteins playing essential roles in metabolic pathways, particularly within the central nervous system (CNS). It participates in excitatory and inhibitory synaptic neurotransmission, with its concentration regulated by transporter-mediated uptake, synthesis, and degradation (Danysz and Parsons 1998; Mizzi and Blundell 2025). Glycine is synthesized primarily through the enzymatic conversion of serine-by-serine hydroxymethyltransferase (SHMT), which operates in both the cytosol and mitochondria, catalyzing the reversible transfer of a hydroxymethyl group from serine to tetrahydrofolate (THF), producing glycine and 5,10-methylene-THF. This pathway is the main quantitative route for endogenous glycine production in humans and is particularly active in tissues with high metabolic rates and demands for nucleotide synthesis, such as the liver and central nervous system. Additional (but secondary) contributions come from the metabolism of choline (via betaine, dimethylglycine, and sarcosine), as well as minor sources like hydroxyproline (Wang et al. 2013; Imenshahidi and Hossenzadeh 2022). The alternative route involves choline metabolism, where choline is sequentially converted into betaine, dimethylglycine, sarcosine, and finally glycine through the action of specific enzymes (Wang et al. 2013; Imenshahidi and Hossenzadeh 2022). Additionally, glycine degradation occurs via the glycine cleavage system (GCS), a mitochondrial enzyme complex predominantly found in astrocytes, which regulates glycine homeostasis and metabolic fluxes in the CNS (Aprison 1990; Danysz and Parsons 1998; Hernandes and Troncone 2009).

As a neurotransmitter, glycine has a dual function in the central nervous system, acting in inhibitory neurotransmission while also modulating excitatory signaling. Its inhibitory action occurs through strychnine-sensitive glycine receptors (GlyRs), contributing to motor control, sensory processing, and reflex modulation, primarily in the brainstem, spinal cord, and cerebellar regions (Aragón and López-Corcuera 2005; Raiteri 2024; Mizzi and Blundell 2025). In contrast, glycine (and D-serine) can facilitate excitatory neurotransmission by serving as an obligatory co-agonist at NMDA receptors, thereby influencing receptor gating, synaptic plasticity, and downstream signaling in cortical and hippocampal circuits. Beyond these established mechanisms, emerging, yet still limited, evidence suggests that glycine may also produce depolarizing effects via glycine-activated excitatory receptors (eGlyRs), including GluN1/GluN3-containing NMDA receptor assemblies that can be activated by glycine in a glutamate-independent manner. In addition, recent reports have proposed the existence of metabotropic glycine receptors (mGlyRs), exemplified by the G-protein-coupled receptor GPR158, which would engage slower, non-ionotropic intracellular signaling cascades to modulate neuronal excitability (Bossi et al. 2023; Laboute et al. 2023). Collectively, these non-canonical glycine signaling modalities remain preliminary and require further replication, mechanistic dissection, and in vivo validation to establish their physiological relevance and translational implications (Bossi et al. 2023; Laboute et al. 2023). The functional distinction between these pathways is critical for maintaining the excitatory-inhibitory balance in the CNS (Stroebel et al. 2021; Raiteri 2024).

The regulation of synaptic glycine levels is mediated by GlyT1 and GlyT2, high-affinity glycine transporters, which co-transport Na + and Cl- ions. While GlyT1 expression is localized mainly, but not exclusively, in glial cells and coupled with glutamatergic excitation, GlyT2 is predominantly, but not exclusively, neuronal and serves as a key component of inhibitory glycinergic pathways. These transporters ensure precise neurotransmitter homeostasis and modulate synaptic efficacy (Betz et al. 2001, Harsing Jr et al. 2006, Bridges et al. 2008, Hernandes and Troncone 2009).

GlyT1 and GlyT2 are critical regulators of central glycine homeostasis and thereby shape both inhibitory glycinergic transmission and NMDA receptor–dependent excitatory signaling. In particular, GlyT1, enriched in perisynaptic astrocytic processes and in selected neuronal compartments at glutamatergic synapses, controls extracellular glycine availability and modulates occupancy of the NMDA receptor co-agonist site, linking glycine dynamics to synaptic plasticity and cognitive function (Zafra et al. 1995a; Marques et al. 2020). Consistent with this role, modulation in glycinergic signaling has been related to benefits against symptoms in a broad spectrum of neurological and psychiatric conditions, including epilepsy, schizophrenia, cerebral ischemia, and neurodegenerative disorders. Accordingly, GlyT1 has emerged as a compelling therapeutic target, and multiple selective GlyT1 inhibitors have been developed to enhance NMDA receptor function by increasing synaptic glycine. These agents have been investigated across indications such as schizophrenia, bipolar disorder, Alzheimer’s disease, neuropathic pain, and sleep-related disorders, with the goal of improving symptom domains linked to glutamatergic dysfunction (Bridges et al. 2008; Salceda 2022). This review highlights the molecular characteristics, physiological roles, and pharmacological relevance of GlyT1, emphasizing its contribution to CNS homeostasis and its potential as a therapeutic target (Danysz and Parsons 1998; Mallorga et al. 2003). A deeper understanding of glycine’s interaction with glutamatergic neurotransmission could contribute to novel therapeutic interventions for CNS disorders.

## Glycine transporters

### Structure

Glycine transporters belong to the SLC6 transporter family, which includes transporters for neurotransmitters such as norepinephrine, dopamine, serotonin, and GABA, as well as orphan homologs with unidentified substrates and bacterial orthologs. These transporters co-transport Na⁺ and Cl⁻ along with their substrates, a mechanism common in neurotransmitter transporters (Raiteri and Raiteri 2010; Rudnick et al. 2014; Cioffi 2018).

GlyT1, encoded by the SLC6A9 gene, and GlyT2, encoded by the SLC6A5 gene, mediate glycine uptake through a conserved transport mechanism involving Na⁺ and Cl⁻ ions. Although GlyT1 and GlyT2 share approximately 50% sequence identity, they exhibit distinct pharmacological properties and tissue distributions. GlyT1 expression is predominantly in glial cells and is associated with excitatory glutamatergic transmission, whereas GlyT2 is primarily found in glycinergic neurons and is critical for inhibitory neurotransmission (Adams et al. 1995; Zhang et al. 2021).

**Fig. 1:**
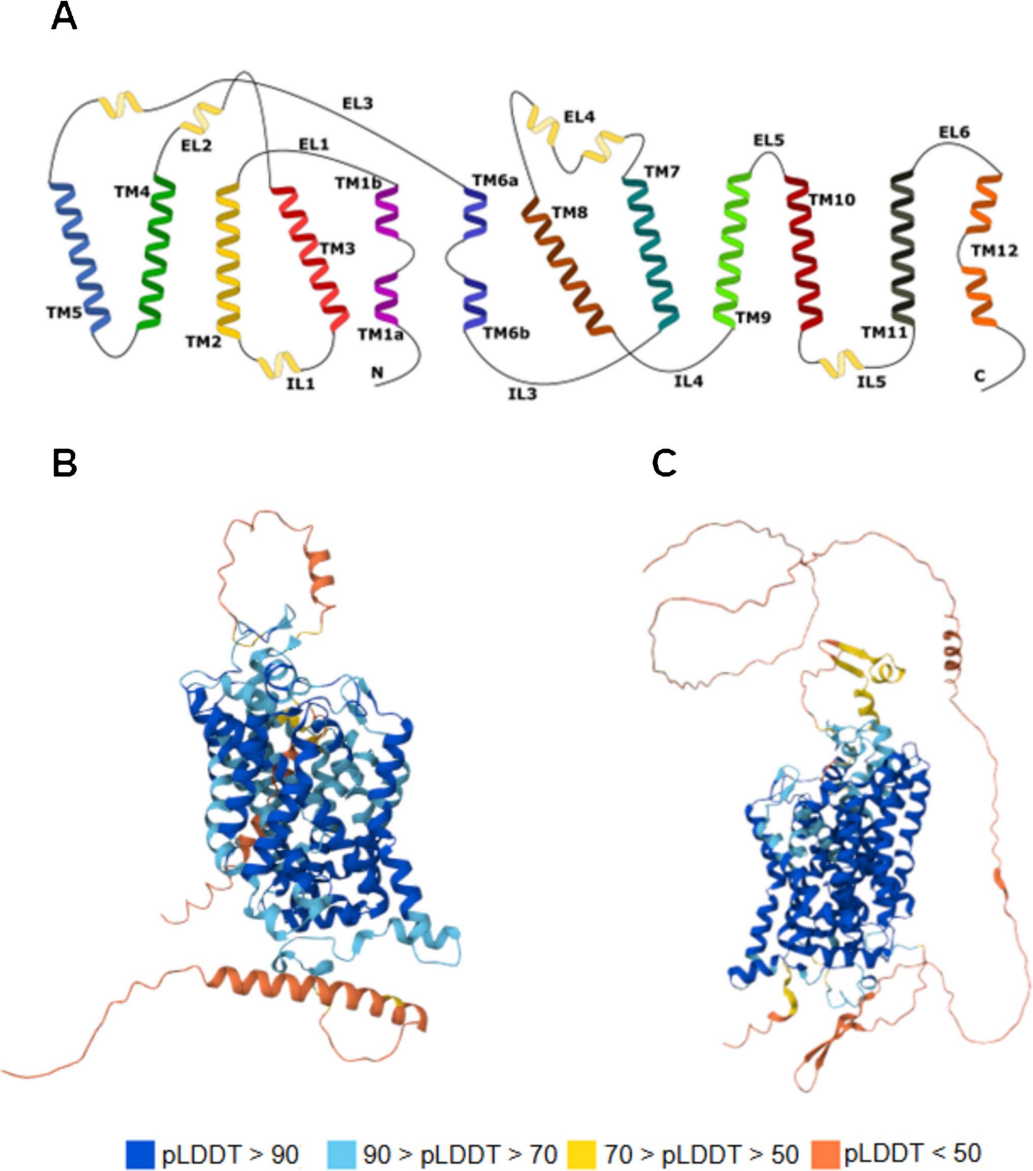
Glycine transporters. **A** A schematic representation of the membrane topology of SLC6, which is characterized by 12 transmembrane domains connected by six hydrophilic extracellular loops between hydrophobic domains 1–2, 3–4, 5–6, 7–8, 9–10, and 11–12. These proteins also feature five intracellular loops between hydrophobic domains 2–3, 4–5, 6–7, 8–9, and 10–11. Structure prediction of **B** GlyT1/SLC6A9 and **C** GlyT2/SLC6A5 (Alphafold v2.3.2; pLDDT; predicted local distance difference test)

The topology of the glycine transporters, GlyT1 and GlyT2, includes 12 transmembrane domains connected by extracellular and intracellular loops (Fig. 1). Notably, the second extracellular loop is highly glycosylated, while transmembrane domains 1 and 3 facilitate substrate passage. Studies have shown that both the N-terminal and C-terminal domains of GlyT1 are located intracellularly and are not essential for transport activity, and all GlyT1 splice variants maintain highly similar transport properties and inhibitor sensitivity (Betz et al. 2001; Aragón and López-Corcuera 2003; Jayaraman et al. 2021; Shahsavar et al. 2021).

Members of the SLC6 transporter family operate according to the alternating-access model (Forrest and Rudnick 2009; Rudnick et al. 2014). Transport is mediated by a “rocking-bundle” mechanism involving two principal structural domains: a mobile “bundle” domain (transmembrane helices 1, 2, 6, and 7) that undergoes conformational changes to permit substrate and ion entry and release and a relatively stationary “scaffold” domain (transmembrane helices 3–5 and 8–10) that provides structural support and guides bundle movements (Forrest and Rudnick 2009; Rudnick et al. 2014; Wei et al. 2024). Despite conservation of the core fold among SLC6 transporters, subtype-specific structural features confer distinct functional profiles.

GlyT1 can operate in reverse mode, allowing glycine efflux under specific conditions, whereas GlyT2 is more efficient at maintaining intracellular glycine levels. Additionally, sarcosine serves as a substrate for GlyT1 but does not interact with GlyT2, highlighting key pharmacological differences between these transporters (Zafra et al. 1995b; Shahsavar et al. 2021; Karpycheva et al. 2025; Li et al. 2025).

The crystallization of human GlyT1, along with structural studies of homologous bacterial and *Drosophila* transporters, has provided insights into the molecular dynamics of glycine transport (Wei et al. 2024). GlyT1 has emerged as a therapeutic target for modulating NMDA receptor signaling across a range of neurological and psychiatric disorders; therefore, its structure has been extensively studied and elucidated to achieve a better understanding of its functions (Webb and Lynch 2007; Herdon et al. 2010; Erdem et al. 2019; Shahsavar et al. 2021).

### Distribution

**Fig. 2:**
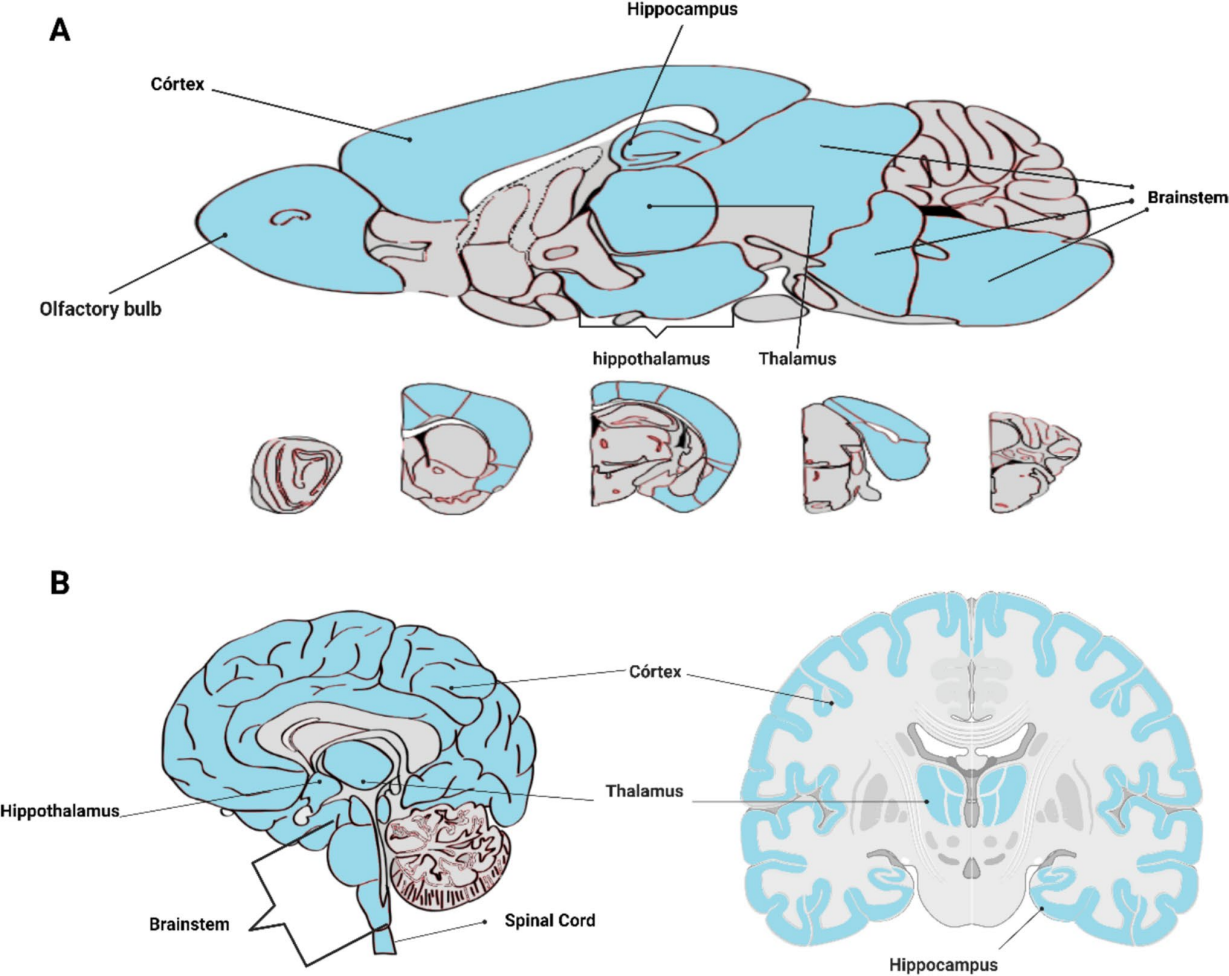
Neuroanatomical distribution of glycine transporter type 1 (GlyT1) in the mouse (**A**) and human (**B**) brains. **A** Schematic illustration of GlyT1 localization in the mouse brain. **B** Schematic illustration of GlyT1 distribution in the human brain. Both panels highlight the major regions expressing GlyT1. Although expression is comparatively lower in areas such as the cortex and hippocampus, these regions remain highly relevant to the investigation of neurodegenerative and neuropsychiatric disorders, as discussed in this review. Adapted from the Human Protein Atlas and created with BioRender

The distinct neuroanatomical localization of GlyT1 and GlyT2 is relevant to their respective functions in both inhibitory and excitatory signaling (Zafra et al. 1995b; Jursky and Nelson 1996; Cioffi 2018). GlyT1 is primarily localized in astrocytes surrounding glycinergic and glutamatergic synapses and is widely distributed throughout the CNS. Morphological mapping studies show that GlyT1 is broadly expressed in glial cells throughout the central nervous system, with higher levels in the hypothalamus, thalamus, olfactory bulb, brainstem, and spinal cord. GlyT1 is also present in forebrain regions, including the neocortex and hippocampus, although at lower levels than in more caudal structures. In the present review, the emphasis on cortical and hippocampal regions reflects their central relevance to glutamatergic neurotransmission and to the pathophysiology of neurodegenerative and neuropsychiatric disorders, rather than the sites of highest absolute GlyT1 expression (Fig. 2). GlyT2 is linked to glycinergic neurons in the spinal cord, brainstem, and cerebellum. It is predominantly localized in presynaptic terminals, where it is linked to high intracellular concentrations of glycine and is primarily involved in inhibitory neurotransmission (Guastella et al. 1992; Liu et al. 1992; Zafra et al. 1995a; Jursky and Nelson 1996; López‐Corcuera et al. 1998; Cioffi 2018).

Regarding expression in different cell types, GlyT1 and GlyT2 have been shown to be expressed at the transcript and protein levels in neurons and astrocytes across a wide range of brain structures, reflecting their functional versatility within the central nervous system. Besides its glial role, GlyT1 can be found in both presynaptic and postsynaptic domains of glutamatergic neurons, where it exhibits spatial co-localization with NMDA receptors. Conversely, GlyT2 displays a more restricted pattern, being primarily localized to presynaptic terminals. Notably, in the hindbrain, GlyT1 is expressed predominantly in glial cells (astrocytes). There, it terminates inhibitory glycinergic transmission by clearing synaptic glycine and modulates glutamatergic signaling by regulating glycine availability at NMDA receptor co-agonist sites (Aroeira et al. 2014; Lopez-Corcuera et al. 2017).

**Fig. 3:**
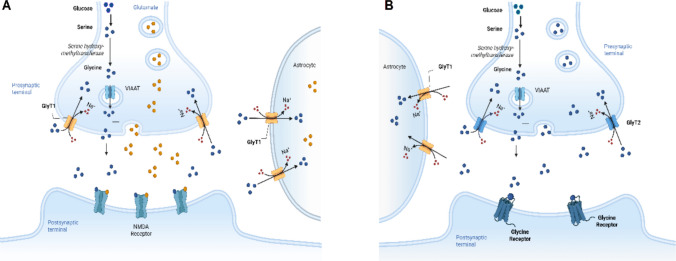
Dual role of glycine in excitatory and inhibitory neurotransmission. **A** In excitatory synapses, glycine acts as a co-agonist at NMDA receptors. It is synthesized from serine-by-serine hydroxymethyltransferase and may be present in glutamatergic terminals, where it can potentially be co-released with glutamate, although this mechanism remains under investigation. In the synaptic cleft, extracellular glycine levels are regulated primarily by GlyT1 transporters located in astrocytes and, to a lesser extent, in presynaptic terminals. Activation of postsynaptic NMDA receptors requires the simultaneous binding of glutamate and glycine, enabling calcium (Ca^2^⁺) influx and excitatory signaling. **B** In inhibitory synapses, glycine serves as a major inhibitory neurotransmitter. It is loaded into synaptic vesicles by VIAAT (vesicular inhibitory amino acid transporter) and released from presynaptic terminals. GlyT2 transporters in presynaptic neurons and GlyT1 transporters in astrocytes are responsible for glycine reuptake and recycling. Postsynaptic glycine receptors, which are chloride (Cl⁻) channels, mediate inhibitory neurotransmission by promoting Cl⁻ influx and hyperpolarization of the postsynaptic membrane

Glial cells specialized for glycine uptake exhibit a high affinity for this neurotransmitter and employ an electrogenic mechanism dependent on the Na⁺ electrochemical gradient and Cl⁻. This mechanism is managed by the Na⁺-K⁺-ATPase, which maintains the Na⁺ gradient in the plasma membrane, providing the energy necessary for neurotransmitter reuptake against concentration gradients and ensuring efficient neurotransmission. In astrocytes, GlyT1 is the most important glycine transporter and is important for regulating glycine levels at inhibitory and excitatory synapses that are essential for neurotransmission mediated by NMDA and GlyR receptors (López‐Corcuera et al. 1998; Zafra and Gimenez 2008; Lopez-Corcuera et al. 2017). Several studies suggest that glycine may be present in a subset of glutamatergic presynaptic terminals, supporting the possibility of glycine–glutamate co-transmission in specific neuronal populations. Anatomical and biochemical analyses have detected glycine in excitatory terminals across distinct brain regions, indicating that it may be co-stored with, or released alongside, glutamate (Zafra et al. 1995a; Bergeron et al. 1998). In addition, electrophysiological evidence indicates that synaptic glycine availability can be modulated by glutamatergic neuronal activity, thereby contributing to the regulation of NMDA receptor activation (Bergeron et al. 1998; Papouin et al. 2012) (Fig. 3).

Two distinct glycinergic pathways have been proposed to account for glycine’s dual actions in the CNS. The first pathway involves strychnine-sensitive glycine receptors, which mediate inhibitory postsynaptic potentials and are found in the brainstem, cerebellum, and spinal cord. The second pathway is characterized by strychnine-insensitive glycine-binding sites that co-localize with NMDA receptors, thereby regulating excitatory glutamatergic synapses in the hippocampus and cortex. Although the second pathway is more directly associated with GlyT1, GlyT1 activity also contributes to the inhibitory pathway (Zafra et al. 1995a; Shahsavar et al. 2021).

### Function

In the CNS, glycine transporters regulate extracellular and synaptic glycine availability, shaping inhibitory transmission through glycine receptors (GlyRs) and modulating NMDA receptor (NMDAR) function by controlling occupancy of the glycine/D-serine co-agonist site (Harsing et al. 2012a, b; Cioffi 2018; Marques et al. 2020; Bae et al. 2021). GlyT1 and GlyT2 fulfill distinct, complementary roles. GlyT1, expressed predominantly in astrocytes and, in a circuit-dependent manner, also in neuronal compartments, acts as a key “buffer” for ambient glycine, thereby setting the gain of NMDAR signaling and limiting glycine spillover. GlyT1 is a Na^+^/Cl^−^ dependent, electrogenic transporter that couples glycine uptake to transmembrane ionic gradients (classically, 1 glycine with 2 Na^+^ and 1 Cl^−^, ultimately maintained by the Na^+^/K^+^-ATPase). Once internalized, glycine can be routed to metabolic pathways (e.g., serine hydroxymethyltransferase–linked one-carbon metabolism) and antioxidant homeostasis (e.g., glutathione synthesis). Although transporter reversal is possible in principle, net glycine release via reverse transport is expected mainly under extreme conditions (e.g., profound depolarization and/or collapse of ionic gradients) and may contribute to maladaptive excitatory cascades rather than constituting a canonical physiological release mechanism (Bridges et al. 2008; Herdon et al. 2010; Raiteri and Raiteri 2010; Raiteri 2024).

GlyT1 and GlyT2 have different transport properties. GlyT1 operates via the co-transport of sodium and chloride ions along with glycine, adapting its function according to local ionic conditions, while GlyT2 is more concentrative (stronger driving force). GlyT2 is specialized for maintaining high presynaptic glycine, at the expense of a greater reliance on ionic gradients (Adams et al. 1995; Lopez-Corcuera et al. 2001; Wang et al. 2022). At the cellular level, GlyT1 transporters mediate glycine uptake from the extracellular space into glial and neuronal cells, influencing synaptic activity and modulating both GABAergic and glutamatergic neurotransmission (Cubelos et al. 2005; Raiteri et al. 2008). While glycine transport is essential for CNS function, the mechanisms governing the synthesis and degradation of glycine transporters remain incompletely understood (Zafra et al. 1995b; Stroebel et al. 2021).

GlyT1 is expressed predominantly, but not exclusively, in glial cells and is also present in glutamatergic neurons, supporting the view that a major function of GlyT1 is to regulate extracellular glycine levels at synapses and thereby modulate NMDAR activity. Glycine acts as an obligatory co-agonist at the GluN1 subunit of NMDARs, which are essential for synaptic plasticity, learning, and memory. By controlling extracellular glycine concentrations, GlyT1 prevents excessive NMDAR activation that could lead to excitotoxicity while balancing glycine levels and maintaining the receptor function and glutamatergic neurotransmission; this modulation is important for maintaining the inhibitory and excitatory balance signaling in the CNS (Borowsky et al. 1993; Adams et al. 1995; Gabernet et al. 2005; Bae et al. 2021; Supplisson 2025).

Given these functions, GlyT1 has been explored as a therapeutic target for drug development, particularly in psychiatric and neurological disorders. GlyT1 inhibitors increase the availability of extracellular glycine, thereby prolonging NMDA receptor activation and affecting synaptic plasticity, memory, and learning. Thus, GlyT1-mediated glycine transport plays a dual role, with the two distinct glycinergic pathways operating in an equilibrium to finely tune glycine’s effects in the CNS. Although glycine has high affinity for the NMDA receptor co-agonist binding site, substantial evidence indicates that this site is not fully saturated under physiological conditions in several brain regions (Bergeron et al. 1998). Electrophysiological studies in cortical and hippocampal neurons have shown that exogenous glycine or D-serine can further potentiate NMDA receptor–mediated currents, indicating that endogenous co-agonist concentrations remain below saturating levels at least in a subset of synapses (Wilcox et al. 1996; Martina et al. 2004). However, these observations are derived mainly from brain-slice and in vitro preparations, which are typically maintained in artificial cerebrospinal fluid devoid of glycine. As a result, the physiological in vivo occupancy of NMDA receptor glycine-binding sites remains uncertain. Importantly, despite the inherent limitations of ex vivo and in vitro models, emerging in vivo and translational evidence supports the view that extracellular glycine availability dynamically regulates NMDA receptor function. For example, SLC6A20 has been identified as a regulator of brain glycine homeostasis, and changes in its activity significantly influence NMDA receptor signaling (Bae et al. 2021). Moreover, recent advances using the brain-penetrant PET tracer [18F]ALX5406 have enabled in vivo assessment of GlyT1 occupancy, further reinforcing the physiological relevance of glycine transport in modulating NMDA receptor function (Hoffmann et al. 2021).

In this context, GlyT1 plays a key role in controlling extracellular glycine levels and, consequently, in modulating NMDA receptor activation. By limiting glycine availability in the synaptic and perisynaptic space, GlyT1 helps maintain submaximal occupancy of the co-agonist binding site, providing a mechanistic basis for the pharmacological inhibition of this transporter as a strategy to enhance NMDA receptor function. GlyT1 inhibitors including bitopertin, NFPS, and sarcosine have been studied as potential therapeutic agents aimed at enhancing NMDA receptor activation and improving cognitive and motor deficits associated with neurodegenerative conditions (Sur and Kinney 2004, 2007). GlyT1 modulation by these inhibitors, selectively blocking glycine reuptake and increasing synaptic glycine concentrations influencing NMDA receptors, makes them promising candidates for treating schizophrenia, Alzheimer’s disease, neuropathic pain, and cerebral ischemia where glutamatergic signaling and glycine homeostasis are important (Zafra et al. 1995b, Sur and Kinney 2004, Aragón and López-Corcuera 2005, Harsing Jr et al. 2006). However, significant challenges persist, including the need to enhance selectivity, ensure safety, and elucidate the long-term effects on neural function. Despite these obstacles, GlyT1 pharmacology remains a compelling area of research, holding promise for the development of novel therapeutic strategies targeting neurological and psychiatric disorders with currently limited treatment options (Harsing Jr et al. 2006, Bridges et al. 2008, Harsing et al. 2012a, b, Pinna and Pałasz 2025).

## Glycine transporter type 1 inhibitors

Glycine transporter inhibitors are a class of pharmacological agents that modulate glycine uptake through specific synaptic transporters in the central nervous system (Cioffi and Guzzo, 2016). Among these, GlyT1 has attracted particular interest in both biomedical research and drug development because of its close functional relationship with NMDA receptor (NMDAR) signaling, which is disrupted in several psychiatric disorders. By increasing extracellular glycine levels, GlyT1 inhibitors can enhance NMDAR function in a regulated manner, thereby improving physiological receptor signaling and synaptic plasticity while potentially avoiding excessive receptor overstimulation. Functionally, GlyT1 occupies a central position in the interplay among glutamatergic, glycinergic, dopaminergic, and possibly other neurotransmitter systems, making it a relevant therapeutic target in a range of neurological and psychiatric conditions under active investigation (Sur and Kinney 2004, Harsing Jr et al. 2006, Bridges et al. 2008, Cioffi 2018).

**Fig. 4:**
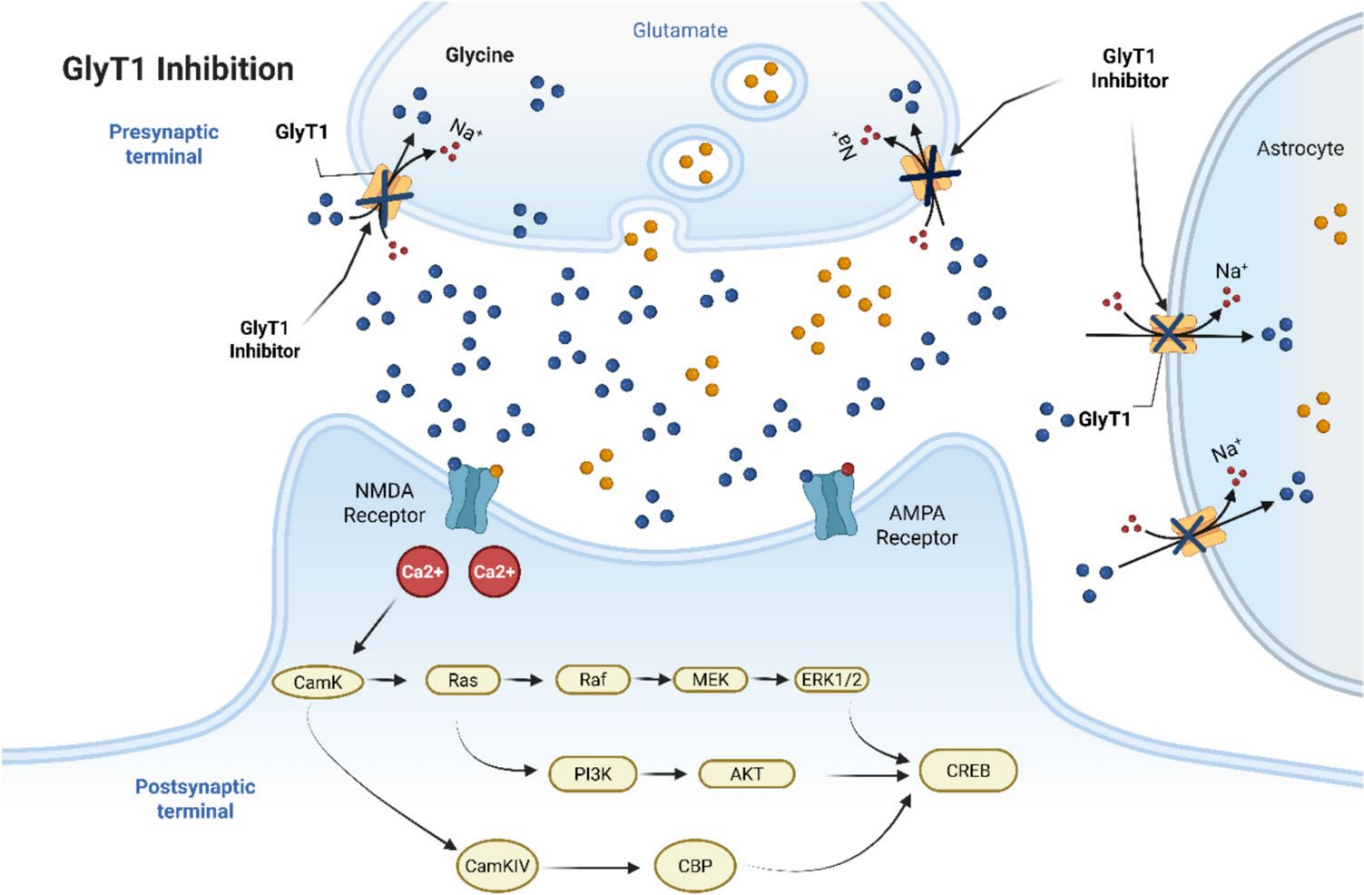
Glutamatergic neurotransmission following the blockade of type 1 glycine transporters (GlyT1). The blockade of GlyT1 increases extracellular glycine concentrations at the synaptic cleft, modulating NMDA receptor activation and enhancing the activation of specific subtypes of this receptor associated with neuronal survival pathways

GlyT1 inhibition increases extracellular glycine availability at synaptic sites, thereby facilitating co-agonist occupancy at NMDA receptors and modulating glutamatergic neurotransmission. In this context, selective inhibition of GlyT1 in forebrain regions has been associated with improvements in both motor and cognitive performance (Fig. 4) (Singer et al. 2007). Moreover, GlyT1 inhibitors have been investigated as potential therapies for neurological, neurodegenerative, and psychiatric disorders including Alzheimer’s disease, schizophrenia, cerebral ischemia, and Parkinson’s disease based on evidence that dysfunctional NMDAR signaling may play an etiological role in these conditions (Cioffi and Guzzo 2016; Marques et al. 2020). Consequently, pharmacological modulation of NMDAR activity through GlyT1 inhibition represents a promising strategy for intervention, although further research is required to clarify the underlying mechanisms of action and to assess the long-term therapeutic effectiveness and tolerability of these compounds (Bridges et al. 2008; Harsing et al. 2012a, b; Marques et al. 2020).

Research on GlyT1 inhibitors emerged in the 1990 s alongside the growing interest in NMDA receptor hypofunction as a key mechanism in schizophrenia. The underlying rationale was that selective inhibition of GlyT1 could increase extracellular glycine availability and thereby enhance NMDA receptor (NMDAR) function. Importantly, this strategy was not conceived as a simple global amplification of glutamatergic signaling. Rather, GlyT1 inhibitors are thought to modulate NMDAR activity in a context- and circuit-dependent manner. By increasing glycine availability at the NMDAR co-agonist site, GlyT1 inhibition may improve receptor function, restore interneuron activity, and rebalance excitation and inhibition within affected neural circuits. In this framework, the pharmacological effects of GlyT1 inhibition may contribute to the normalization of glutamatergic neurotransmission not only in hypoglutamatergic states but also in conditions where network dysfunction secondarily coexists with excessive glutamatergic tone. This mechanistic perspective has supported the investigation of GlyT1 inhibitors in schizophrenia and has also encouraged interest in their potential application to certain neurodegenerative disorders (Lechner 2006; Harsing et al. 2012a, b; Cioffi 2018).

These findings were met with enthusiasm, as GlyT1 inhibition demonstrated promise in ameliorating specific symptoms and cognitive deficits in some neurodegenerative conditions (Bridges et al. 2008; Yang and Svensson 2008). Consequently, multiple research groups launched drug discovery programs targeting GlyT1, giving rise to a highly competitive field marked by structurally diverse chemotypes and a large number of patent filings. Subsequent studies showed that several GlyT1 inhibitors were effective in preclinical models predictive of antipsychotic and procognitive effects, including models relevant to Alzheimer’s disease and cerebral ischemia. Some of these compounds subsequently advanced to clinical trials for schizophrenia (Yang and Svensson 2008; Dohi et al. 2009; Wolkenberg and Sur 2010).

**Fig. 5:**
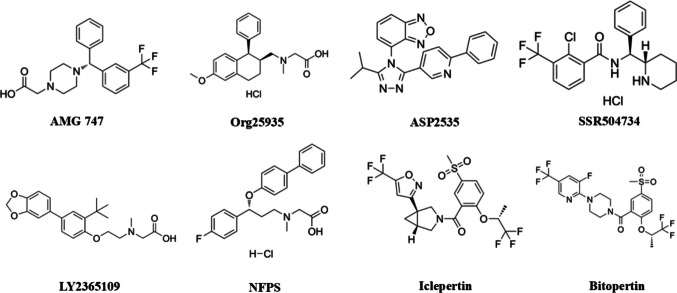
Chemical structures of known GlyT1 inhibitors. The figure illustrates a diverse panel of compounds that have been classified as inhibitors of the type 1 glycine transporter (GlyT1), including AMG 747, Org25935, ASP2535, SSR504734, LY2365109, NFPS, iclepertin, and bitopertin. These molecules vary in chemical scaffolds and binding affinities and have been investigated for their potential therapeutic applications in neurological, psychiatric, and neurodegenerative disorders

GlyT1 inhibitors can be broadly classified into two categories: sarcosine-derived and non-sarcosine-derived compounds. Notable GlyT1 inhibitors include sarcosine, the glycine derivative NFPS [N-[3-(4′-fluorophenyl)−3-(4′-phenylphenyl)propyl]-N-methylglycine], and bitopertin (Fig. 5). These agents have demonstrated potential in preclinical models and clinical trial studies for modulating glycinergic neurotransmission, suggesting their potential as therapeutic strategies for a variety of neurological and psychiatric disorders. However, it remains critical to mention that glycine transporter inhibitors may be associated with adverse effects and complications, such as gastrointestinal disturbances and systemic toxicity. The following discussion provides relevant background information on each structural category, contextualizing the associated intellectual property.

### Sarcosine

Sarcosine, also referred to as *N*-methylglycine, is an endogenous amino acid involved in glycine and one-carbon metabolism. It is generated from glycine by glycine *N*-methyltransferase and can be converted back to glycine through the action of sarcosine dehydrogenase. In addition, sarcosine is functionally connected to dimethylglycine metabolism, further highlighting its participation in intermediary metabolic pathways (Mallorga et al. 2003; Zhang et al. 2009b). From a pharmacological perspective, sarcosine is considered a relatively weak GlyT1 inhibitor, with reported IC_50_ values in the low millimolar range (approximately 1–3 mM), indicating that high concentrations are required to achieve substantial inhibition of glycine transport (Zhang et al. 2009b). The development of more potent GlyT1 inhibitors was initially inspired by structural modifications of sarcosine, particularly through the introduction of bulky hydrophobic substituents at the nitrogen atom, an approach guided by emerging structural and pharmacological insights into GlyT1 and its relationship to other members of the SLC6 transporter family.

Over the past decades, sarcosine has attracted increasing attention in biomedical research, particularly in the context of neuropsychiatric and neurodegenerative disorders. This interest is largely supported by evidence indicating that sarcosine can modulate glycinergic and glutamatergic neurotransmission, two systems critically involved in synaptic plasticity, neuronal survival, and higher-order brain function (Tsai et al. 2004; Zhang et al. 2009a, b; Pinto et al. 2012, 2014; Pan et al. 2022). Through these mechanisms, sarcosine has emerged as a molecule of translational interest in central nervous system disorders characterized by synaptic dysfunction, excitatory-inhibitory imbalance, and progressive neuronal damage.

In schizophrenia, sarcosine has been investigated mainly as an adjunct to antipsychotic treatment, with several studies reporting beneficial effects on symptom severity and functional outcomes (Lin et al. 2017; Kumar et al. 2023). These observations are consistent with the hypothesis that enhancement of glycine-dependent signaling may mitigate aspects of NMDA receptor hypofunction, a mechanism widely implicated in the pathophysiology of schizophrenia (Zhang et al. 2009a).

In parallel, preclinical studies have suggested that sarcosine may exert neuroprotective effects in neurodegenerative and acute neurological injury models. In the 5XFAD model of Alzheimer’s disease, for example, sarcosine treatment has been reported to attenuate β-amyloid plaque burden and improve cognitive performance, suggesting a potential influence on disease-related processes associated with synaptic failure and memory decline (Pan et al. 2022). Likewise, in experimental models of cerebral ischemia, sarcosine has shown neuroprotective activity in both in vivo and in vitro settings, further expanding its potential relevance beyond chronic neurodegeneration to acute brain injury (Pinto et al. 2012, 2014). Collectively, these studies indicate that sarcosine may have therapeutic value across distinct neurological contexts, although the underlying mechanisms are likely multifactorial and not exclusively attributable to GlyT1 inhibition. Despite these limitations, sarcosine remains an attractive research candidate with potential implications for the development of future therapeutic strategies in central nervous system disorders (Socała et al. 2010; Pinto et al. 2012, 2014; Marques et al. 2020).

### NFPS

N-[3-(4′-fluorophenyl)−3-(4′-phenylphenoxy)propyl] sarcosine (NFPS), also known as ALX-5407, is a sarcosine-based GlyT1 inhibitor. Due to its lipophilic nature, NFPS tends to accumulate within the plasma membrane, diffusing through the lipid bilayer until it reaches GlyT1. Available evidence indicates that NFPS binds to an extracellular site on GlyT1, with findings suggesting a preferential interaction with the outward-facing conformation of the transporter. Other lipophilic GlyT1 inhibitors derived from sarcosine may share a similar mechanism of action. In contrast to sarcosine, NFPS is not transported by GlyT1, which characterizes it as a non-transportable inhibitor. This feature is particularly relevant because it contributes to its distinct pharmacological profile. NFPS demonstrates an approximately 100-fold greater efficacy than sarcosine and exhibits high affinity for GlyT1, with an IC_50_ in the range of 5–10 nM. In addition, NFPS displays slow dissociation kinetics, consistent with a pseudo-irreversible mode of interaction with the transporter and is therefore considered a highly potent and selective GlyT1 inhibitor. Consequently, unlike sarcosine, NFPS does not induce glycine efflux when administered alone. However, it effectively antagonizes the glycine-releasing effects elicited by sarcosine or glycine. Kinetic analyses further indicate that NFPS inhibits GlyT1 activity in a non-competitive manner, a property that has also been confirmed for its active enantiomer, ALX-5407. Several studies have demonstrated beneficial effects of NFPS in neurodegenerative conditions, including Parkinson’s disease and cerebral ischemia (Atkinson et al. 2001; Aubrey and Vandenberg 2001b; Harsing Jr et al. 2003).

### Bitopertin

Bitopertin, a compound belonging to the benzoylpiperazine class, was developed by Roche as part of an effort to identify highly selective GlyT1 inhibitors with improved pharmacokinetic properties and enhanced safety profiles. It was discovered through high-throughput screening followed by lead optimization and was subsequently characterized as a potent and selective inhibitor of the GlyT1 transporter, with an IC_50_ of 30–40 nM (Pinard et al. 2018). Also known as RG1678, bitopertin was the first non-sarcosine-derived GlyT1 inhibitor to be evaluated in preclinical models of schizophrenia and to advance into phase II and phase III clinical trials. This compound exhibits potent and selective non-competitive inhibition of the GlyT1 subtype, without significant effects on other molecular targets (Umbricht et al. 2014). Bitopertin has been extensively investigated in both preclinical and clinical studies aimed at the development of therapeutic strategies for schizophrenia; however, it ultimately failed to meet the primary endpoints in phase III trials. Nevertheless, the literature has also documented neuroprotective effects of bitopertin in other pathological conditions, including Parkinson’s disease and neuropathic pain (Pinard et al. 2018; Frouni et al. 2022).

### Iclepertin

Iclepertin (BI 425809) is a GlyT1 inhibitor developed by Boehringer Ingelheim after the discontinuation of bitopertin, representing a continued effort to therapeutically target GlyT1. It has been investigated for improving cognitive deficits associated with schizophrenia and is reported to be a highly selective, reversible inhibitor, with an IC_50_ of 10–20 nM, currently in advanced clinical development for cognitive disorders (Rosenbrock et al. 2018; Harvey et al. 2025). Phase II and phase III studies have evaluated the efficacy, safety, and clinical potential of iclepertin for cognitive dysfunction in schizophrenia (Rosenbrock et al. 2022). In phase II, iclepertin was generally well tolerated and was associated with significant cognitive improvements. However, more recent phase III findings indicated that iclepertin did not achieve significant improvements in cognitive or functional outcomes compared with placebo after six months of treatment. Despite this, the drug was associated with minimal adverse effects and good overall tolerability (Schultheis et al. 2022; Reuteman-Fowler et al. 2024; Harvey et al. 2025). Overall, iclepertin remains a therapeutic candidate for cognitive impairment in schizophrenia and potentially for neurodegenerative conditions, with ongoing and future trials expected to further clarify its efficacy and safety profile.

### Other GlyT1 inhibitors

Currently, several studies are focused on the identification and characterization of novel GlyT1 inhibitors, such as Amgen’s benzhydryl piperazine AMG 747, Organon’s Org25935 and Org24461, NPTS, ASP2535, CP-802,079, PF-03463275, SSR504734, LY2365109, and BI 425809. Several of these compounds have progressed to clinical evaluation, known for their potency and selectivity as a GlyT1 inhibitor, progressed to phase II clinical trials, particularly focusing on schizophrenia aimed at assessing their efficacy as an adjunctive therapy for negative symptoms in schizophrenic patients receiving concurrent antipsychotic treatment, but these trials were discontinued several years ago due to unspecified safety concerns (Javitt et al. 1994; Javitt 2012). Significant advancements have been achieved in recent years in the identification and development of potent and selective GlyT1 inhibitors by various academic and industrial research teams covering a range of structural classes. To simplify the literature, it is useful to categorize compounds into those containing sarcosine, such as NFPS, and those lacking sarcosine. A notable trend observed in the literature is a shift from sarcosine-based to non-sarcosine-based inhibitors over time (Brown et al. 2001; Legendre 2001; Cioffi and Guzzo 2016). Given the availability of several comprehensive reviews and original studies addressing both sarcosine-derived and non-sarcosine-derived compounds, the current body of evidence indicates that GlyT1 inhibitors may exert therapeutically relevant effects in neurodegenerative diseases (Nishikawa et al. 2010; Cioffi 2018; Marques et al. 2020).

## Patents of GlyT1 inhibitors

In the last decade, several research groups have focused on the discovery and development of new GlyT1 inhibitors and have investigated their potential therapeutic utility in various neurodegenerative diseases. Most recent applications and patents now pertain to the non-sarcosine-based class of inhibitors. For example, Albany Molecular Research, Inc. developed and patented a new series of GlyT1 inhibitors structurally based on piperazine scaffolds via a rational design approach that tethered secondary benzamide and sulfonamide moieties (Cioffi et al. 2013, 2016). In 2015 and 2018, Abbott detailed the preparation and biological assessment of aminotetraline, aminochromane, and isoindoline scaffolds, representing emerging classes of competitive GlyT1 inhibitory agents (Amberg et al. 2016, 2018). Additionally, AbbVie, Inc. has explored new GlyT1 inhibitor analogs, including pyrrolidine and 4,5-dihydropyrrazole derivatives (Korablina et al. 2016; Amberg et al. 2017). Furthermore, research on the solid forms of GlyT1 inhibitors remains an active area; Boehringer Ingelheim International has focused on methods for producing solid forms, developing pharmaceutical compositions that include these forms, and evaluating their use in treating medical conditions responsive to GlyT1 inhibition.

GlyT1 inhibitors have garnered increasing attention due to their therapeutic potential in neurological and psychiatric disorders. The modulation of the glutamatergic system, with which GlyT1 is intricately linked, represents a promising strategy for treating various conditions. GlyT1 inhibitors show potential for the treatment of neurodegenerative and psychiatric disorders. Moreover, these inhibitors are being explored for potential therapeutic use in other pathological conditions, including specific hematological disorders (Matte et al. 2019; Taher et al. 2021). However, realizing these therapeutic outcomes will require further advances in drug design, comprehensive clinical studies, and an enhanced understanding of the molecular mechanisms underlying their pharmacological effects.

## Neuropharmacology of GlyT1 inhibition

GlyT1 inhibitors have emerged as potential therapeutic agents for various neurological and psychiatric disorders, given their role in modulating glycinergic neurotransmission. In vivo evaluations of these compounds have been conducted in diverse species, including mice, rats, non-human primates, and chickens, to investigate their effects on conditions such as addiction, aging, Alzheimer’s disease, autism, epilepsy, Huntington’s disease, ischemia, pain, Parkinson’s disease, schizophrenia, and stroke (Cioffi and Guzzo 2016; Marques et al. 2020).

The studies span nearly the last three decades, reflecting the sustained and growing scientific interest in the modulation of glycinergic neurotransmission as a therapeutic strategy. The administered doses vary widely, ranging from nanomolar concentrations to milligrams per kilogram, with treatment regimens encompassing both single-dose administrations and prolonged protocols lasting several weeks. Among the most extensively studied compounds are ALX5407 (NFPS), Bitopertin, Sarcosine, Org24598, Org25935, and SSR504734, demonstrating promising efficacy across multiple pathological contexts.

### Psychiatric disorders

#### Schizophrenia

Schizophrenia constitutes a complex psychiatric syndrome, characterized by disruptions in cognitive processing, increased motor agitation, and exaggerated stereotypical behaviors. Pharmacological interventions targeting NMDA receptors have emerged as promising approaches for managing this condition. According to the glutamate hypothesis, dysfunction of NMDA receptor–mediated neurotransmission has been implicated in the pathophysiology of negative symptoms in schizophrenia (Hu et al. 2015; Pei et al. 2021).

GlyT1 inhibition enhances NMDA receptor signaling by increasing synaptic glycine availability, offering a modulatory alternative to direct NMDA receptor activation and its associated adverse effects (Kemp and Leeson 1993; Kinney et al. 2003; Chue 2013; Pei et al. 2021). Research has examined glycine and GlyT1 as therapeutic targets to modulate glutamatergic neurotransmission and mitigate schizophrenia symptoms, with multiple studies emphasizing their relevance (Javitt et al. 1994; Harada et al. 2012; Harvey and Yee 2013; Dunayevich et al. 2017). Furthermore, forebrain-specific studies have indicated that GlyT1 knockout results in reduced glycine transport activity, enhances glycinergic transmission, and improves NMDA receptor function, suggesting that strengthening NMDA receptor signaling could help counteract cognitive and attentional deficits associated with schizophrenia (Yee et al. 2006).

The development of GlyT1 inhibitors was initially motivated by the search for novel antipsychotic therapies targeting the negative symptoms of schizophrenia, which have been associated with dysregulated glutamatergic neurotransmission (Möhler et al. 2011; Javitt 2012). A seminal study by Aubrey and Vandenberg (2001a) laid the foundation for this field by proposing glycine transport inhibition as a treatment approach focused on restoring NMDA receptor functionality to alleviate core features of schizophrenia (Aubrey and Vandenberg 2001a). Given the hypothesized dysfunction of the NMDA receptor in schizophrenia, efforts to enhance glycinergic neurotransmission through GlyT1 inhibition gained traction as a therapeutic strategy.

Different GlyT1 inhibitors have been synthesized and assessed in preclinical models. Notably, ALX5407 (NFPS) has shown effectiveness in attenuating hyperlocomotion in phencyclidine-induced models of schizophrenia (Aubrey and Vandenberg 2001b; Harsing Jr et al. 2003). However, its effects in amphetamine-induced models have been inconsistent, with some studies reporting no significant effects (Harsing Jr et al. 2003). Another GlyT1 inhibitor, Org24461, exhibited similar efficacy in PCP-induced hyperactivity models but failed to show benefits in amphetamine-induced models (Harsing Jr et al. 2003).

Several novel GlyT1 inhibitors, including SSR103800 and SSR504734, have demonstrated potential therapeutic effects in schizophrenia, with positive evidence from experimental models addressing multiple schizophrenia-relevant domains, such as psychotic-like behavior, social withdrawal, and cognitive impairment (Black et al. 2009). SSR504734, in particular, has been evaluated in DBA/2 mice, NMDA-mediated current assays, and amphetamine-induced hyperactivity models, demonstrating promising results (Depoortère et al. 2005). SSR103800 demonstrated efficacy in improving episodic memory and object recognition in rodent paradigms, highlighting its relevance to cognitive symptom domains (Boulay et al. 2008).

ASP2535 has also demonstrated efficacy in PCP- and MK-801-induced schizophrenia models, improving cognitive deficits in preclinical studies (Harada et al. 2012). Other inhibitors such as TASP0315003 have shown promising findings from preclinical PCP-induced schizophrenia models further supporting the therapeutic relevance of GlyT1 inhibition (Chaki et al. 2015). The translation of these findings into clinical applications has led to multiple trials assessing the potential of GlyT1 inhibitors to ameliorate symptoms of schizophrenia. Bitopertin (RG1678) initially demonstrated efficacy in schizophrenia patients by modulating NMDA receptor function (Alberati et al. 2012). However, while early-phase trials reported favorable findings, later studies yielded mixed results, with some phase II trials failing to show significant improvement in negative symptoms (Umbricht et al. 2014; Bugarski-Kirola et al. 2016). Sarcosine, another GlyT1 inhibitor tested as an adjunct to atypical antipsychotics, showed beneficial effects in phase II trials, particularly in improving overall clinical symptoms, but meta-analyses indicate no substantial impact on cognitive function (Lane et al. 2010).

Recent investigations indicate that GlyT1 inhibitors, including BI 425809 (iclepertin), exhibit therapeutic efficacy in preclinical models of schizophrenia, particularly in mitigating MK-801-induced hyperactivity (Rosenbrock et al. 2022). Clinical trials of BI 425809 have yielded encouraging results in phase I and phase II studies, reinforcing its potential cognitive benefits in schizophrenia (Moschetti et al. 2018; Schultheis et al. 2022). However, a 2025 phase II randomized, double-blind, placebo-controlled trial did not confirm these findings, indicating the necessity for further validation (Harvey et al. 2025).

Additionally, AMG 747, another GlyT1 inhibitor, demonstrated therapeutic effects at a dose of 15 mg; however, this effect was not observed at doses either above or below this effective range. These findings are consistent with earlier reports describing an inverted U-shaped dose–response relationship, underscoring the importance of further investigation into the pharmacodynamic profile of GlyT1 inhibitors. In this context, the continued evaluation of these compounds as potential treatments for the negative symptoms of schizophrenia remains a critical priority (Dunayevich et al. 2017). The development and clinical implementation of GlyT1 inhibitors for the treatment of schizophrenia have advanced significantly, supported by robust preclinical and clinical data (Table 1). However, additional research and extended clinical trials are necessary to fully elucidate the benefits and potential limitations of this pharmacological strategy. While compounds such as bitopertin, BI 425809, and SSR103800 have demonstrated potential in early-stage clinical and preclinical studies, translating these findings into viable treatments remains complex, necessitating further in-depth investigations (Javitt 2012).

**Table 1:** GlyT1 inhibition in psychopharmacology

Year	Drug	Doses	Species	Model	References
*Schizophrenia models*					
2001	ALX5407	10 mg/kg	Rat	Phencyclidine (PCP)-induced model	(Atkinson et al. 2001)
2003	Org24461	3.8 mg/kg	Mouse	PCP- and D-amphetamine-induced hypermotility	(Harsing Jr et al. 2003)
2008	SSR103800	0.5–3 mM	Rat	NMDA-mediated currents	(Boulay et al. 2008)
2008	SSR103800	10–30 mg/kg	Mouse	GMO-DBA/2 mice	(Boulay et al. 2008)
2008	SSR103800	20–30 mg/kg	Mouse	MK-801-induced hyperactivity	(Boulay et al. 2008)
2008	SSR103800	10–30 mg/kg	Rat	Phencyclidine (PCP)-induced model	(Boulay et al. 2008)
2008	SSR103800	0.1–10 mg/kg	Rat	Phencyclidine (PCP)-induced model (Neonatal)	(Boulay et al. 2008)
2008	SSR103800	30 mg/kg	Mouse	Prepulse inhibition (PPI) of startle reflex	(Boulay et al. 2008)
2008	SSR103800	1–10 mg/kg	Rat	Amphetamine-induced model	(Boulay et al. 2008)
2008	SSR103800	1–10 mg/kg	Rat	MK-801-induced hyperactivity	(Boulay et al. 2008)
2010	SSR103800	10–30 mg/kg	Mouse	GMO-NMDA Nr1neo −/− transgenic mouse	(Boulay et al. 2010)
2010	SSR103800	10–30 mg/kg	Mouse	MK-801-induced hyperactivity	(Boulay et al. 2010)
2010	ALX5407	3–20 mg/kg	Mouse	GMO-DBA/2 mouse model	(Kopec et al. 2010)
2010	Merk (S)−13h	3–20 mg/kg	Mouse	DBA/2 mouse model	(Kopec et al. 2010)
2010	Roche-7	30–300 mg/kg	Mouse	DBA/2 mouse model	(Kopec et al. 2010)
2010	Sarcosine	1000–6000 mg/kg	Mouse	DBA/2 mouse model	(Kopec et al. 2010)
2010	Sarcosine	2 g/day	Human	Phase II (add-on treatment to atypical antipsychotics)	(Lane et al. 2010)
2011	Org24461	16 mg	Human	Phase II (ketamine-induced psychosis)	(D’souza et al. 2012)
2011	RG1678	0.3–3 mg/kg	Mouse	Amphetamine-induced model	(Alberati et al. 2012)
2011	RG1678	0.3–300 nM	Rat	NMDA receptor modulation	(Alberati et al. 2012)
2011	RG1678	1–10 mg/kg	Rat	Phencyclidine (PCP)-induced model	(Alberati et al. 2012)
2011	SSR‑504,734	3–10 mg/kg	Rat	Attentional set-shifting task (ASST)	(Nikiforuk et al. 2011)
2012	ASP2535	0.3–3 mg/kg	Rat	MK-801-induced hyperactivity	(Harada et al. 2012)
2012	ASP2535	0.1–3 mg/kg	Mouse	Phencyclidine (PCP)-induced model	(Harada et al. 2012)
2012	ASP2535	0.3–3 mg/kg	Rat	Phencyclidine (PCP)-induced model	(Harada et al. 2012)
2012	ASP2535	0.1–3 mg/kg	Rat	Scopolamine-induced model	(Harada et al. 2012)
2012	ASP2535	0.1–3 mg/kg	Rat	Aged rats	(Harada et al. 2012)
2013	RG1678	0.3–1.0 mg/kg	Monkey	Scopolamine-induced model	(Eddins et al. 2014)
2014	RG1678	10–60 mg/d	Human	Phase II (patients with schizophrenia)	(Umbricht et al. 2014)
2015	TASP0315003	0.3–10 mg/kg	Rat	Phencyclidine (PCP)-induced model	(Chaki et al. 2015)
2016	AMG 747	15 mg	Human	Phase II (enduring negative symptoms)	(Dunayevich et al. 2017)
2016	RG1678	3–60 mg	Human	Phase I (healthy volunteers)	(Hofmann et al. 2016)
2016	RG7118	15–30 mg	Human	Phase I (healthy volunteers)	(Hofmann et al. 2016)
2018	BI 425809	0.5–150 mg	Human	Phase I (healthy volunteers)	(Moschetti et al. 2018)
2018	BI 425809	10–75 mg	Human	Phase I (healthy volunteers)	(Moschetti et al. 2018)
2018	BI 425809	0.5–150 mg	Human	Phase II (patients with schizophrenia)	(Moschetti et al. 2018)
2022	BI 425809	0.3, 1, 4 mg/kg	Mouse	MK-801-induced hyperactivity	(Rosenbrock et al. 2022)
2022	BI 425809	2–25 mg	Human	Phase II: patients with schizophrenia	(Schultheis et al. 2022)
2023	Sarcosine	300 or 600 mg/kg, i.p	Rat	Ketamine-induced model	(Kumar et al. 2023)
2025	BI 425809	10 mg	Human	Phase II: patients with schizophrenia	(Harvey et al. 2025)
*Addiction models*					
2011	Org 24,598	12 mg/kg	Rat	Ethanol consumption	(Lidö et al. 2012)
2012	RO4543338	30–45 mg/kg	Rat	Cocaine-seeking behavior	(Dhonnchadha et al. 2012)
2012	Org 24,598	3–7.5 mg/kg	Rat	Cocaine self-administration	(Achat-Mendes et al. 2012)
2012	Org 24,598	1 mg/kg	Monkey	Cocaine self-administration	(Achat-Mendes et al. 2012)
2013	SSR504734	10 mg/kg	Rat	Nicotine-seeking behavior	(Cervo et al. 2013)
2017	Org 24,598	3–6 mg/kg	Rat	Ethanol consumption	(Lido et al. 2017)
2022	Org 24,598	0.3–1.0 mg/kg	Rat	Ethanol consumption	(Filarowska-Jurko et al. 2022)
2024	Org 24,598	6–9 mg/kg	Rat	Ethanol consumption	(Olsson et al. 2024)

#### Addiction

Substance abuse encompassing alcohol, cocaine, methamphetamine, and heroin can trigger addiction by disrupting multiple neural pathways and initiating complex neuromodulatory processes that alter behavior. A significant challenge in treating drug addiction is the persistent influence of environmental cues associated with substance use, which continue to drive drug-seeking behavior. Several pharmacological and genetic strategies targeting GlyT1 inhibition have demonstrated efficacy in numerous animal models of drug-seeking behavior (Table 1) (M Cleva et al. 2010; Harvey and Yee 2013). In this context, GlyT1 inhibition shows promise for modulating NMDA receptor function in dopaminergic neurons, thereby potentially altering the reward circuitry associated with addiction (Schmitz et al. 2013).

Neurochemical and molecular dysregulations observed in the brains of affected individuals with drug addiction predominantly affect dopaminergic transmission within limbic and reward systems (Carlezon Jr and Thomas 2009; Harhai and Harsing 2022). Scientific investigations have emphasized the promise of GlyT1 inhibition as a treatment strategy, particularly Org 24,598, in mitigating substance abuse, with preclinical studies demonstrating its anti-addictive properties in multiple addiction paradigms. Notably, Org 24,598 has demonstrated the capacity to reduce cocaine self-administration in non-human primates following intravenous administration at 1.0 mg/kg across three experimental sessions, as well as in rodent models at intraperitoneal doses ranging from 3.0 to 7.5 mg/kg, supporting its role in attenuating cocaine-seeking behavior (Achat-Mendes et al. 2012). Additionally, the GlyT1 inhibitor RO4543338, administered during extinction training, was effective in reducing cocaine-seeking behavior and response to cocaine-associated cues in rats at doses of 30–45 mg/kg (i.p.) over a 3-week period (Dhonnchadha et al. 2012).

Similarly, the GlyT1 inhibition has been investigated for its potential to reduce alcohol consumption. Org 24,598 significantly reduced alcohol consumption and preferences in rats when administered during a 2-week treatment period (12 mg/kg, i.p.) (Lidö et al. 2012) and the same GlyT1 inhibitor (0.3–1.0 mg/kg, i.p.) was effective in reversing ethanol-induced behavioral impairments (Filarowska-Jurko et al. 2022). Moreover, Org 24,598 administered prior to alcohol exposure (6–9 mg/kg, i.p.) over 7 days in rats resulted in reduced voluntary alcohol consumption (Olsson et al. 2024). Similar effects were observed for its structural analog, Org25935, which also attenuated alcohol consumption in rats at equivalent doses (3–6 mg/kg, i.p.) (Lidö et al. 2017). Beyond alcohol and cocaine, the role of GlyT1 inhibition has been investigated in relation to nicotine-seeking behavior. SSR504734, administered post-session at 10 mg/kg (i.p.), significantly reduced nicotine-seeking in rats following either a single-dose or a 5-day treatment regimen (Cervo et al. 2013).

### Neurological disorders

#### Epilepsy

Epilepsy, characterized by sudden and repetitive episodes of sensory disturbances, loss of consciousness, or convulsions due to abnormal electrical activity, results from an imbalance of neurotransmitters and neuromodulators in the brain (Akyuz et al. 2021). Glycinergic transmission in conjunction with GABAergic signaling and NMDA receptor function is fundamental for maintaining neuronal excitability homeostasis. Dysfunction in glycine receptor activity has been associated with epileptiform discharges, and it has been proposed that changes in GlyT1 expression within the hippocampus may underlie key aspects of the disorder’s neurochemical dysfunction, and the resulting imbalance in glycine homeostasis may contribute to the pathophysiology of epilepsy (Rigo et al. 2002; Chen et al. 2014; Boison 2016). Recent findings highlight the potential of GlyT1 inhibitors as modulators of seizure susceptibility, where GlyT1 inhibition maintains the balance between excitatory and inhibitory signals in the brain that are often disrupted in epilepsy (Shen et al. 2015; Cioffi 2018).

NFPS (ALX5407) administered to rats in a maximal electroshock threshold (MEST) model showed positive effects, increasing the seizure threshold with doses ranging from 1.0 to 32 mg/kg (p.o.) (Kalinichev et al. 2010). GSK931145, tested in the same MEST model, also exhibited dose-dependent anticonvulsive effects (1.0–10 mg/kg, p.o.) (Kalinichev et al. 2010). Similarly, Lu AA21279, another GlyT1 inhibitor, demonstrated a positive effect in the MEST test at doses of 3.0–30 mg/kg (p.o.) (Kalinichev et al. 2010). Another GlyT1 inhibitor tested was Org25935; at doses of 3.0–30 mg/kg in MEST models, it produced a robust increase in seizure threshold (Kalinichev et al. 2010). Moreover, SSR504734, another potent GlyT1 inhibitor, demonstrated positive results in MEST models across various dose levels (3.0–30 mg/kg, p.o.) with positive effects observed in rats and mice (Kalinichev et al. 2010). SSR504734 in another study also showed anticonvulsive effects in the PTZ model in a single-dose regimen, although the effects were not consistent across all models tested (Gapińska et al. 2025).

In the MEST model, sarcosine administered at doses of 100–800 mg/kg (i.p.) demonstrated positive anticonvulsant effects (Socała et al. 2010). Moreover, in the timed intravenous pentylenetetrazole (PTZ) infusion test, sarcosine administration was associated with an elevation in seizure threshold levels, providing further evidence of its anticonvulsive properties (Socała et al. 2010). Sarcosine also has been shown to raise seizure thresholds and suppress kindling epileptogenesis (Shen et al. 2020).

In preclinical models of temporal lobe epilepsy (TLE), LY2365109 was administered to mice and rats. At doses from 3 to 30 mg/kg (i.p.), LY2365109 significantly increased seizure thresholds and suppressed chronic seizures, indicating its potential for managing epilepsy (Shen et al. 2015). This effect was similarly observed in rats with doses of 7.5–30 mg/kg (i.p.) (Shen et al. 2015). GlyT1 inhibitor M22 treatment increased the seizure threshold in a mouse model of the maximal electroshock seizure threshold (MEST) test without affecting motor function, suggesting its potential as a new anticonvulsive drug but in this same study M22 was ineffective in altering the seizure threshold as assessed by the PTZ infusion test (Zhao et al. 2016).

This experimental evidence supports the notion that GlyT1 inhibition such as NFPS, SSR504734, M22, sarcosine, and LY2365109 exhibits promising anticonvulsant effects in several epilepsy models (Table 2). These compounds raised seizure thresholds, suppressed chronic seizures, and demonstrated neuroprotective effects through mechanisms involving glycine receptor modulation. However, studies also reported null effects on certain models. For instance, M22 and SSR504734 administered in the PTZ model showed no significant effects, indicating that the anticonvulsant properties of GlyT1 inhibitors might be model-dependent (Zhao et al. 2016; Gapińska et al. 2025). The lack of efficacy in certain experimental setups underlines the need for further research to optimize treatment regimens and assess the long-term safety and GlyT1 inhibitors' effectiveness in their applications for epilepsy treatment.

**Table 2:** GlyT1 inhibition in neuropharmacology

Year	Drug	Doses	Species	Model	References
*Epilepsy models*					
2010	ALX5407	1–32 mg/kg	Rat	Maximal electroshock threshold	(Kalinichev et al. 2010)
2010	GSK931145	1–10 mg/kg	Rat	Maximal electroshock threshold	(Kalinichev et al. 2010)
2010	Lu AA21279	3–30 mg/kg	Rat	Maximal electroshock threshold	(Kalinichev et al. 2010)
2010	Org25935	3.0–30 mg/kg	Rat	Maximal electroshock threshold	(Kalinichev et al. 2010)
2010	SSR504734	3–30 mg/kg	Rat	Maximal electroshock threshold	(Kalinichev et al. 2010)
2010	SB710622	1.6–32 mg/kg	Rat	Maximal electroshock threshold	(Kalinichev et al. 2010)
2010	Sarcosine	100–800 mg/kg	Mouse	Maximal electroshock threshold	(Socała et al. 2010)
2015	LY2365109	3–30 mg/kg	Mouse	Temporal lobe epilepsy (TLE)	(Shen et al. 2015)
2015	LY2365109	7.5–30 mg/kg	Rat	Temporal lobe epilepsy (TLE)	(Shen et al. 2015)
2016	M22	10–40 mg/kg	Mouse	Maximal electroshock threshold	(Zhao et al. 2016)
2020	Sarcosine	3 g/kg	Rat	Epileptogenesis	(Shen et al. 2020)
2025	SSR504734	10–50 mg/kg	Mouse	Maximal electroshock threshold	(Gapińska et al. 2025)
*Neuropathic pain models*					
2008	ALX5407	10–100 µg	Rat	Chronic constriction injury (CCI)	(Hermanns et al. 2008)
2008	Org25935	3–300 ng	Mouse	Partial sciatic nerve ligation	(Morita et al. 2008)
2008	Org25935	3–300 ng	Mouse	STZ-induced diabetic model	(Morita et al. 2008)
2008	Sarcosine	20 ng–0.3 mg/kg	Mouse	Partial sciatic nerve ligation (PSNI)	(Morita et al. 2008)
2008	Sarcosine	20 ng–0.3 mg/kg	Mouse	STZ-induced diabetic model	(Morita et al. 2008)
2014	ALX5407	0.2–200 μg/kg	Rat	Chronic constriction injury (CCI)	(Barthel et al. 2014)
2018	RG1678	10 mg/kg	Mouse	Inflammatory pain	(Armbruster et al. 2018)
2018	RG1678	2 mg/kg	Mouse	Neuropathic pain	(Armbruster et al. 2018)
2018	RG1678	2 mg/kg/day	Mouse	Neuropathic pain	(Armbruster et al. 2018)
2018	RG1678	1 mg/kg	Rat	Neuropathic pain	(Armbruster et al. 2018)
2021	ALX5407	1–4 mg/kg	Rat	Partial sciatic nerve ligation	(Mohammadzadeh et al. 2021)

#### Neuropathic pain

Neuropathic pain is a chronic painful condition caused by damage to the nervous system, often resulting in allodynia and hyperalgesia, which are difficult to treat with conventional medications. The use of GlyT1 inhibitors has emerged as a promising therapeutic approach, as a result of their capacity to modulate glycinergic inhibitory transmission within the spinal cord, a key region involved in nociceptive processing (Dohi et al. 2009).

Studies with ALX5407, a selective GlyT1 inhibitor, have shown consistent results in neuropathic pain preclinical models induced by chronic constriction injury (CCI) (Hermanns et al. 2008; Barthel et al. 2014). In this 2008 study, intrathecal (i.t.) administration of ALX5407 (doses of 10, 50, and 100 µg) resulted in significant analgesic effects, with an increase in mechanical pain thresholds observed up to 240 min after administration. This study demonstrated the GlyT1 inhibitor’s efficacy in restoring glycinergic inhibition, providing relief from neuropathic pain (Hermanns et al. 2008).

In a later study in 2014, ALX5407 was administered subcutaneously via osmotic pump (0.2, 2, 20, 200 μg/kg/day), resulting in positive effects over a 14-day period in Wistar rats (Barthel et al. 2014). This study corroborated previous findings and suggested that continuous administration of ALX5407 could produce prolonged analgesic effects without significant adverse effects on motor activity or general behavior (Barthel et al. 2014). Continuous modulation of glycine concentrations in cerebrospinal fluid (CSF) was relevant for the therapeutic efficacy.

In additional models, such as partial sciatic nerve ligation (PSNL) and streptozotocin-induced diabetic models (STZ), GlyT1 inhibitors such as Org25935 and sarcosine proved effective in attenuating neuropathic pain. Intrathecal administration of Org25935 and sarcosine (doses ranging from 3 to 300 ng for Org25935 and 20 to 300 ng for sarcosine) led to a significant increase in both mechanical and thermal pain thresholds in mice, with positive effects observed for up to 10 days post-administration. These effects were attributed to the increase in glycine concentrations in the CNS, which contribute to the modulation of pain through the activation of glycine receptors (Morita et al. 2008).

More recent studies, such as those conducted with NFPS in 2021, reinforce the therapeutic potential of selective GlyT1 inhibitors in the treatment of neuropathic pain. Subcutaneous administration of NFPS (1, 2, 4 mg/kg) in rats with sciatic nerve injury (PSNL) resulted in analgesic effects in both acute and chronic treatments (4 days). This inhibitor at subanalgesic doses also significantly increased pain thresholds, supporting the hypothesis that modulating glycinergic neurotransmission can alleviate neuropathic pain without adversely affecting motor function (Mohammadzadeh et al. 2021). Another study investigated the effect of the specific GlyT1 inhibitor, bitopertin (RG1678), in models of neuropathic and inflammatory pain in mice and rats. Bitopertin improved response thresholds to thermal and mechanical stimuli without altering motor activity or affecting acute pain processing (Armbruster et al. 2018).

The accumulated evidence indicates that GlyT1 inhibitors such as ALX5407 and ORG25935 represent an innovative and potential therapeutic approach for managing neuropathic pain (Table 2). These compounds promote increased extracellular glycine in the central nervous system, leading to the recovery of spinal inhibitory glycinergic function, which is important for pain control. Both continuous and single-dose administration have shown clinical efficacy, with a favorable safety profile. Future clinical trials will be essential to validate the effectiveness and safety of these compounds in humans, potentially opening new avenues for managing chronic painful conditions.

#### Cerebral ischemia

Cerebral ischemia, a cerebrovascular event caused by the obstruction of blood flow, induces brain injury and cognitive deficits. The ischemic process depletes cellular energy reserves, such as phosphocreatine and adenylate, while also promoting cellular acidification due to the accumulation of lactic acid from anaerobic glycolysis during tissue hypoxia (Lee et al. 2018; Zhang et al. 2022). Additionally, ischemia triggers excessive glutamate release, leading to overactivation of NMDA receptors, which in turn causes excitotoxicity, oxidative stress, and neuroinflammation. This scenario struggles to maintain ion homeostasis, resulting in abnormal depolarization and increased neurotransmitter release (Gidday 2006; Liu et al. 2009). Excitotoxicity severity is influenced by NMDA receptor subunit composition and activation, with GluN2A and GluN2B exhibiting different responses to excitotoxicity (Choo et al. 2012; Zhao et al. 2022). NMDA receptors containing GluN2B exacerbate NMDA-induced excitotoxicity; this association suppresses the neuroprotective effects of survival cell pathways and initiates intracellular signaling pathways leading to cell death. Conversely, GluN2A-containing NMDA receptors can activate pro-survival signaling pathways, including ERK and CREB phosphorylation, and enhance BDNF (Chen et al. 2015; Zhang et al. 2015; Ladagu et al. 2023).

Recent research has highlighted the involvement of glycine in neuroprotective mechanisms during cerebral ischemia. GlyT1 inhibition modulation in NMDA receptor activity has been investigated for its potential neuroprotective effects against ischemic insults (Cappelli et al. 2022; Qin et al. 2022). Low-dose glycine treatment following middle cerebral artery occlusion (MCAO) in rodent models has demonstrated neuroprotective effects, reducing infarct volume and neurological deficits (Lu et al. 2012). In contrast, high-dose glycine treatment in a permanent MCAO model increased brain damage, due to differential activation of NMDA receptor subtypes. Glycine at low concentrations appears to promote neuroprotection by engaging CREB signaling cascades downstream of GluN2A-subunit-containing NMDA receptor activation (Chen et al. 2015; Zhang et al. 2015).

Several studies have demonstrated the protective glycine and GlyT1 inhibitors’ effects in experimental ischemia models. Pretreatment with the GlyT1 inhibitors, sarcosine (competitive) and NFPS/ALX5407 (non-competitive), has been shown to promote neuroprotection in both ex vivo oxygen–glucose deprivation (OGD) models and in vivo global cerebral ischemia models (Pinto et al. 2012, 2014, 2015; Cavalcante et al. 2024). The neuroprotective GlyT1 inhibitors’ effects have been associated with a reduction in GluN2B-containing NMDA receptor expression and modulation of glycinergic and glutamatergic neurotransmission (Pinto et al. 2015).

In rodent MCAO models, NFPS at high doses (6 mg/kg, post-treatment) significantly reduced infarct volume and neuronal injury, along with decreased cleaved caspase-3 expression and increased Bcl-2/Bax ratio, indicating reduced apoptosis and enhanced cell survival (Huang et al. 2016). Notably, NFPS also improved spatial learning deficits in rats subjected to MCAO, further supporting its potential benefits for cognitive recovery post-ischemia (Huang et al. 2016). A recent study using MCAO in C57 mice found that a single pre-treatment with NFPS 24 h before ischemia significantly reduced infarct size and improved motor deficits, reinforcing that GlyT1 inhibition enhances neuroprotection by increasing GluN2A expression while decreasing GluN2B expression (Cavalcante et al. 2024).

In addition to NFPS, sarcosine has shown efficacy in global cerebral ischemia models. Pre-treatment with sarcosine (300–500 mg/kg, i.p.) for 7 days before global ischemia (4VO model) in rats resulted in significant neuroprotection, as evidenced by reduced neuronal loss, infarct volume, and cognitive impairment (Pinto et al. 2014). Similarly, in hippocampal OGD models, sarcosine (30–300 mg/kg, pre-treatment) conferred neuroprotection by reducing excitotoxic damage (Pinto et al. 2012).

Supporting the therapeutic relevance of GlyT1 blockade in ischemic models, ex vivo experiments have demonstrated that NFPS and similar inhibitors showed significant neuroprotection in OGD-induced ischemic models. NFPS (10–100 µM) failed to show significant neuroprotection in hippocampal OGD models, but it exhibited positive effects in retinal OGD models (10 µM, rat; 0.3 mM, chicken) (Tanabe et al. 2010; Harsing Jr et al. 2012; Hanuska et al. 2016). Similarly, Org-24461 and TBOA demonstrated protective effects in retinal ischemia models, reinforcing the idea that GlyT1 inhibition may enhance neuronal resilience under ischemic conditions (Harsing Jr et al. 2012).

Moreover, emerging research suggests that GlyT1 inhibition plays a role in preserving vascular integrity in ischemic conditions. Studies indicate that glycine-mediated NMDA receptor modulation may promote vasculature stability in the peri-infarct region, further contributing to its protective effects (Cappelli et al. 2022). Pre-administration of NFPS prior to NMDA-induced excitotoxic insult in mouse models (1.25–5 mg/kg, i.p.) has been shown to reduce neuronal damage, reinforcing its potential role in limiting ischemia-induced injury (Pinto et al. 2015).

These findings collectively suggest that targeting GlyT1 inhibition to increase glycine levels could be a promising pharmacological strategy for treating cerebral ischemia (Table 3). By modulating NMDA receptor function, reducing excitotoxicity, and enhancing neuroprotective pathways, GlyT1 inhibitors like NFPS and sarcosine may offer novel therapeutic avenues to mitigate ischemic brain injury and promote functional recovery. However, further research is needed to optimize treatment strategies, define optimal dosing regimens, and evaluate the long-term safety and neuroprotective efficacy associated with GlyT1 inhibition in cerebral ischemia.

**Table 3:** GlyT1 inhibition in neuroprotection

Year	Drug	Doses	Species	Model	References
*Cerebral ischemia model*					
2012	Sarcosine	30–300 mg/kg	Rat	Oxygen/glucose deprivation (OGD)	(Pinto et al. 2012)
2014	Sarcosine	300–500 mg/kg	Rat	Brain ischemia–four-vessel occlusion (4VO)	(Pinto et al. 2014)
2016	Sarcosine	3 mmol/L	Rat	Oxygen/glucose deprivation (OGD)—retina	(Hanuska et al. 2016)
2012	ALX5407	0.3 mM	Chicken	Oxygen/glucose deprivation (OGD)—retina	(Harsing Jr et al. 2012)
2015	ALX5407	1.25–5 mg/kg	Mouse	NMDA-induced excitotoxicity	(Pinto et al. 2015)
2016	ALX5407	6.0 mg/kg	Rat	Middle cerebral artery occlusion (transient)	(Huang et al. 2016)
2016	ALX5407	10 µmol/L	Rat	Oxygen/glucose deprivation (OGD)—retina	(Hanuska et al. 2016)
2024	ALX5407	1.25–5 mg/kg	Mouse	Middle cerebral artery occlusion (permanent)	(Cavalcante et al. 2024)
2012	Org-24461	0.3 mM	Chicken	Oxygen/glucose deprivation (OGD)—retina	(Harsing Jr et al. 2012)
2016	ACPPB	1 µmol/L	Rat	Oxygen/glucose deprivation (OGD)—retina	(Hanuska et al. 2016)
2016	TBOA	10 µmol/L	Rat	Oxygen/glucose deprivation (OGD)—retina	(Hanuska et al. 2016)
*Parkinson’s disease models*					
2013	ACPPB	19.8 ml/min/kg	Mouse	Intrastriatal 6-OHDA injection	(Schmitz et al. 2013)
2021	ALX5407	0.01–1 mg/kg	Marmoset	Parkinsonian by MPTP injection	(Frouni et al. 2021)
2022	RG1678	0.03–3 mg/kg	Rat	6-Hydroxydopamine model	(Frouni et al. 2022)
2024	ALX5407	0.03–3 mg/kg	Rat	6-Hydroxydopamine model	(Frouni et al. 2024)
2024	ALX5407	1.25–5 mg/kg	Mouse	6-Hydroxydopamine model	(Ribeiro et al. 2024)
*Alzheimer’s disease models*					
2012	ASP2535	0.1 mg/kg	Rat	Aged rats	(Harada et al. 2012)
2023	BI 425809	2.0–25.0 mg/kg	Human (phase II)	Patients with Alzheimer	(Wunderlich et al. 2023)
2024	ALX5407 (NFPS)	2.5–5.0 mg/kg	Mouse	Injection of amyloid-β peptide	(Oliveira-Lima et al. 2024)

### Neurodegenerative disorders

#### Parkinson’s disease

The motor symptoms of Parkinson’s disease result from the gradual neurodegeneration affecting dopaminergic cell populations in the substantia nigra pars compacta. This loss leads to an imbalance in the cortico-basal ganglia circuit and disrupts various neurotransmission systems (Schmidt and Thompson 2016). Dyskinesia, a common symptom of Parkinson’s disease, is related to glutamatergic neurotransmission, making its modulation a promising therapeutic target. NMDA receptor activity modulation through GlyT1 inhibition has been associated with producing anti-dyskinetic effects and reducing other parkinsonian symptoms (Olivares et al. 2012).

Research by Frouni and colleagues demonstrated that GlyT1 inhibitors like ALX5407 (NFPS) could alleviate both parkinsonian symptoms and dyskinesia, offering a novel strategy to treat and potentially prevent motor complications in Parkinson’s disease (Frouni et al. 2021). In additional studies, Frouni and colleagues used GlyT1 inhibitors, including NFPS (ALX5407), to investigate GlyT1 distribution in motor-related brain regions such as the cortex, basal ganglia, and thalamus, all of which are implicated in Parkinson’s disease and dyskinesia. Their findings indicated a reduction in GlyT1 expression in the thalamus and subthalamus, suggesting that this downregulation may act as a compensatory defense mechanism. The GlyT1 downregulation in the thalamus would result in increased glycine levels, potentially enhancing glutamatergic transmission at NMDA receptors (Frouni et al. 2024).

Furthermore, a study focused on the striatum, an essential motor area with a role in both cognition and Parkinson’s disease, using a 6-hydroxydopamine (6-OHDA) PD model demonstrated that pretreatment with NFPS provided significant neuroprotection. This treatment reduced neuronal degeneration, preserved dopaminergic neurons, maintained dendritic spines in the striatum region, and led to decreased motor impairments (Ribeiro et al. 2024). Consistent with these findings, bitopertin administered subcutaneously (0.03–3 mg/kg) post-lesion in rats with 6-OHDA-induced Parkinson’s disease provided substantial neuroprotective effects, preserving dopaminergic integrity and improving motor outcomes. When administered over a 4-week period, bitopertin also demonstrated long-term benefits in maintaining motor function (Frouni et al. 2022).

Additional findings underscore the potential contribution of GlyT1 blockade to Parkinson’s disease modulation coming from studies utilizing the MPTP model of parkinsonism in marmosets, where a single post-treatment dose of NFPS (0.01–1 mg/kg, i.p.) significantly attenuated parkinsonian motor deficits (Beaudry and Huot 2020; Frouni et al. 2021). Furthermore, studies using the GlyT1 inhibitor ACPPB in a 6-OHDA mouse model demonstrated neuroprotective effects following intrastriatal injection of 6-OHDA, with ACPPB treatment (19.8 ml/min/kg) administered post-lesion over 4 weeks (Schmitz et al. 2013).

These researchers suggest that GlyT1 inhibition may offer a neuroprotective strategy in Parkinson’s disease by modulating glutamatergic transmission, preserving dopaminergic neurons, and mitigating dyskinesia (Table 3). Future investigations should focus on the long-term safety and efficacy of GlyT1 inhibitors, particularly in combination with existing dopaminergic therapies, to optimize treatment strategies for the patients with Parkinson’s disease.

#### Alzheimer’s disease

Alzheimer’s disease is a neurodegenerative disorder characterized by a progressive cognitive decline and memory impairment. It is one of the most common causes of dementia worldwide, affecting millions of individuals and posing significant public health challenges (Lee et al. 2018; Zhang et al. 2022). N-Methyl-D-aspartate (NMDA) receptor dysfunction participates in various neurological disorders, including AD (Harada et al. 2012; Yoo et al. 2022). This dysfunction is associated with cognitive decline and an increased occurrence of seizures, with irregularities in glutamatergic pathways being a key mechanism. NMDA receptor activity depends on agonists and co-agonists such as glycine for optimal function, and disturbances in glycine availability may contribute to NMDA receptor hypofunction (Harada et al. 2012; Rosenbrock et al. 2018; Yoo et al. 2022).

GlyT1 inhibitors have emerged as a novel class of compounds under investigation for the treatment of cognitive impairment in Alzheimer’s disease. By modulating glycine homeostasis, these compounds indirectly enhance NMDA receptor function, with potential benefits for cognitive performance. For example, research indicates that NMDA receptor signaling, which underlies long-term potentiation and synaptic plasticity, can be enhanced by GlyT1 inhibitors, ASP2535 and BI 425809. These compounds elevate glycine levels in the synaptic cleft, thereby augmenting NMDA receptor activation and potentially enhancing cognitive function and memory (Harada et al. 2012; Rosenbrock et al. 2018; Wunderlich et al. 2023).

GlyT1 inhibition has demonstrated neuroprotective efficacy in preclinical models of Alzheimer’s disease. The GlyT1 inhibitor ALX5407 (NFPS) showed to prevent amyloid-β-induced cognitive deficits when administered 24 h prior to an intrahippocampal injection of amyloid-β in mice, improving both short-term and long-term memory as assessed by novel object recognition and spatial memory tasks (Oliveira-Lima et al. 2024). Similarly, ASP2535 demonstrated cognitive benefits in aged rats when administered orally with a dose of 0.1 mg/kg for 4 days, suggesting its potential for counteracting age-related cognitive decline (Harada et al. 2012).

Despite encouraging preclinical findings, clinical trials have yielded mixed results. BI 425809 has been investigated in a phase II trial involving patients with Alzheimer’s disease, where the compound was administered at doses of 2–25 mg over 12 weeks, failing to demonstrate significant cognitive improvements (Wunderlich et al. 2023). These findings highlight the complexity of NMDA receptor modulation in Alzheimer’s disease and underscore the need for further investigations into the mechanisms by which GlyT1 inhibitors influence disease progression and cognitive function (Table 3).

## Translational limitations

Despite substantial preclinical evidence supporting the neuroprotective and procognitive GlyT1 inhibition neurobiological effects, clinical translation has encountered considerable limitations. The most emblematic example is bitopertin (RG1678), which initially demonstrated efficacy in phase II trials for schizophrenia negative symptoms yet failed to meet primary endpoints in multiple phase III trials (Umbricht et al. 2014; Bugarski-Kirola et al. 2016). These failures have been attributed not only to variability in trial design and patient heterogeneity but also to a potential mismatch between preclinical models and human pathophysiology (Frouni et al. 2022).

Although bitopertin’s selectivity for GlyT1 may not have provided sufficient synaptic glycine elevation in relevant cortical areas or may have induced compensatory mechanisms that attenuated its efficacy, iclepertin (BI 425809), which was developed following the bitopertin setback, showed promise in early trials and even reached phase III (Reuteman-Fowler et al. 2024). However, recent findings indicate that iclepertin did not produce statistically significant cognitive or functional improvements in schizophrenia after 6 months of treatment, despite being safe and tolerated (Rosenbrock et al. 2018; Harvey et al. 2025). These results raise questions about the actual therapeutic window for GlyT1 inhibition, especially regarding duration, dose, and the regional specificity of transporter modulation.

An additional translational challenge is defining the degree of GlyT1 inhibition required to produce clinically meaningful effects. Preclinical evidence suggests that partial transporter inhibition may be sufficient to increase extracellular glycine levels and enhance NMDA receptor activation, whereas excessive inhibition could disturb the finely regulated balance of co-agonist availability and synaptic signaling (Javitt 2012; Castner et al. 2014). Furthermore, the relationship between systemic drug exposure and effective central target engagement remains incompletely understood. Although several GlyT1 inhibitors have shown adequate brain penetration in both preclinical and clinical studies, determining the extent to which these compounds elevate glycine concentrations within specific synaptic microdomains in vivo remains challenging. Progress in this area has been supported by the development of positron emission tomography (PET) tracers that enable assessment of GlyT1 distribution and occupancy in the human brain, such as [^11^C]RO5013853 (Martin-Facklam et al. 2013; Wong et al. 2013). Even so, the number of validated PET ligands remains limited, and establishing a robust relationship between transporter occupancy and functional modulation of NMDA receptor signaling continues to be a major translational obstacle. In parallel, electrophysiological and microdialysis studies indicate that extracellular glycine concentrations are tightly regulated and vary across brain regions and physiological states (Bergeron et al. 1998; Martina et al. 2004; Martin-Facklam et al. 2013). This variability is particularly relevant because the glycine co-agonist site of NMDA receptors is not uniformly saturated under basal conditions, indicating that the pharmacodynamic effects of GlyT1 inhibition are likely to depend on local co-agonist availability within the synaptic and perisynaptic environment.

Another critical, and often underappreciated, consideration is the pharmacological selectivity of GlyT1 inhibitors within the broader SLC6 family, which comprises closely related transporters such as GlyT2 (SLC6A5) and PROT (SLC6A7), as well as several monoamine transporters (e.g., DAT, SERT, and NET) (de Carvalho et al. 2024). While most clinical candidates are described as “selective” for SLC6A9, few have been comprehensively profiled against the full complement of SLC6 proteins (de Carvalho et al. 2024; Nascimento et al. 2026). Even minimal off-target interactions can lead to unintended modulation of neurotransmitter systems, potentially confounding therapeutic outcomes or exacerbating side effects. For instance, partial inhibition of GlyT2 could interfere with inhibitory glycinergic signaling, while residual affinity for SLC6A7 may alter L-proline homeostasis and glutamatergic tone, especially in limbic regions (Bae et al. 2021; de Carvalho et al. 2024).

Moreover, the lack of high-resolution data on the binding kinetics and transport-coupling mechanisms of these inhibitors across different SLC6 members complicates the interpretation of both efficacy and safety findings in vivo (Erdem et al. 2019). This is particularly relevant when considering that expression patterns of SLC6 transporters are region- and cell type–specific, and their physiological roles are tightly interdependent (Bröer and Gether 2012). Thus, the pharmacological isolation of GlyT1 as a therapeutic strategy demands not only high-affinity binding but also rigorous exclusion of functional cross-reactivity within the SLC6 family.

Future development of GlyT1-targeted therapies should prioritize enhanced selectivity at both structural and functional levels. This can be achieved through structure-based drug design and mechanism-informed approaches, such as biased inhibition or allosteric modulation, to fine-tune transporter engagement. An integrative strategy should also incorporate in vivo selectivity assays, transcriptomic co-expression analyses, and disease-contextualized transporter profiling to ensure beneficial neuromodulatory effects while minimizing compensatory or deleterious responses.

## Conclusion

The pharmacological inhibition of GlyT1 has produced significant promise for a wide range of therapeutic applications, particularly in the context of neurological and psychiatric disorders. Over the past years, studies have investigated the implications of GlyT1 modulation across diverse pathological conditions, including substance use disorders, schizophrenia, neurodegenerative diseases, and cognitive impairments.

In addiction models, such as those related to cocaine and ethanol consumption, GlyT1 inhibition consistently showed positive effects. These studies, which involved both pre-treatment and during-treatment dosing regimens, suggest that pharmacological inhibition of GlyT1 has been proposed as a strategy to diminish the rewarding effects of drugs of abuse. Given that these substances influence glutamatergic systems, the ability to modulate glycine transporter activity represents a novel approach for addiction treatment, especially with the encouraging results observed in rats and monkeys.

Additionally, studies conducted in the last 5 years have expanded the therapeutic GlyT1 inhibitors’ focus to neurodegenerative conditions such as Alzheimer’s disease. By elevating extracellular glycine levels, GlyT1 inhibition enhances NMDA receptor–mediated neurotransmission, potentially addressing cognitive deficits and synaptic dysfunction commonly observed in Alzheimer’s and related dementias. This suggests a neuroprotective effect, which could slow the progression of cognitive decline, a fundamental aspect in aging-related neurodegenerative diseases.

Moreover, the potential benefits of GlyT1 inhibition in the schizophrenia treatment continue to be investigated. Schizophrenia is often associated with glutamatergic dysfunction, particularly within the NMDA receptor system. By increasing glycine levels in the synaptic cleft, GlyT1 inhibitors may improve cognitive function and negative symptoms, offering an alternative or adjunct to existing antipsychotic treatments that have limited efficacy in these domains.

Despite the promising results observed in recent studies, several challenges remain in fully harnessing the therapeutic potential of GLYT1 inhibitors. Although these compounds show promise across various therapeutic areas, ongoing research is important to determine the optimal dosing, long-term safety, and the complete spectrum of their efficacy in preclinical and clinical settings. A significant challenge lies in translating preclinical findings to human clinical trials, which require rigorous testing across diverse populations to confirm the clinical GlyT1 inhibition benefits in treating conditions such as schizophrenia, cerebral ischemia, Alzheimer’s disease, and addiction. The accumulated evidence provides a robust foundation that the inhibition of GlyT1 is increasingly recognized as a viable strategy for the treatment of multiple neurological, psychiatric, and neurodegenerative diseases.

## Data Availability

All source data for this work (or generated in this study) are available upon reasonable request.

## Abbreviations

AD: Alzheimer’s disease
ASD: Autism spectrum disorder
CCI: Chronic constriction injury
CNS: Central nervous system
DNA: Deoxyribonucleic acid
GCS: Glycine cleavage system
Glu: Glutamate
GlyR: Strychnine-sensitive glycine receptor
GlyT1: Glycine transporter type 1
GlyT2: Glycine transporter type 2
IPSP: Inhibitory postsynaptic potential
mRNA: Messenger ribonucleic acid
MCAO: Middle cerebral artery occlusion
Na⁺/K⁺-ATPase: Sodium-potassium ATPase
nNOS: Neuronal nitric oxide synthase
NMDAR: N-Methyl-D-aspartate receptor
OGD: Oxygen-glucose deprivation
pCREB: Phosphorylated cAMP response element-binding protein
PD: Parkinson’s disease
PROT: Proline transporter
PSD-95: Postsynaptic density protein 95
SCZ: Schizophrenia
SHMT: Serine hydroxymethyltransferase
SLC: Solute carrier family
SLC6A5: Gene encoding GlyT2
SLC6A7: Gene encoding PROT
SLC6A9: Gene encoding GlyT1
VIAAT: Vesicular inhibitory amino acid transporter

## Declarations

### AI-assisted technologies in the writing process

During the preparation of this manuscript, the author(s) used ChatGPT (version 4.0) solely for language editing and grammar refinement. The tool did not contribute to the scientific content, data interpretation, or idea generation. The author(s) subsequently reviewed and revised the manuscript as needed and took full responsibility for the content of the publication.
